# Supplementary material for: Meta-Analysis and Gene Set Enrichment Relative to ER Status Reveal Elevated Activity of MYC and E2F in the “Basal” Breast Cancer Subgroup
Source: PLoS One. 2009 Mar 9;4(3):e4710. doi: 10.1371/journal.pone.0004710 (PMC2650420; doi:10.1371/journal.pone.0004710)
Supplement: Dataset S1 — GSEA results from first screen. The file contains a zipped archive of the HTML results of the second GSEA screen. Reports for enriched or depleted datasets may be accessed from the “index.html” link within the my_analysis.GseaPreranked.1219975950625.rpt.zip ZIP archive. Enrichment plots are available for each of the 50 most enriched and depleted gene sets. (13.32 MB ZIP) [file pone.0004710.s001.zip › my_analysis.GseaPreranked.1219975950625.rpt.zip!//KANNAN_P53_UP.html]

Details for gene set KANNAN\_P53\_UP[GSEA]

|  || Dataset | zSco.ERneg.to.ERpos\_collapsed |
| Phenotype | NoPhenotypeAvailable |
| Upregulated in class | na\_neg |
| GeneSet | KANNAN\_P53\_UP |
| Enrichment Score (ES) | -0.43189177 |
| Normalized Enrichment Score (NES) | -1.6032463 |
| Nominal p-value | 0.013927577 |
| FDR q-value | 0.12418635 |
| FWER p-Value | 0.999 |
Table: GSEA Results Summary

  

Fig 1: Enrichment plot: KANNAN\_P53\_UP      
 Profile of the Running ES Score & Positions of GeneSet Members on the Rank Ordered List

  

| PROBE | GENE SYMBOL | GENE\_TITLE | RANK IN GENE LIST | RANK METRIC SCORE | RUNNING ES | CORE ENRICHMENT || 1 | EXT2 |  |  | 1103 | 3.355 | -0.0292 | No |
| 2 | ADFP |  |  | 1342 | 3.090 | 0.0025 | No |
| 3 | PCNA |  |  | 1346 | 3.087 | 0.0518 | No |
| 4 | ATF3 |  |  | 3455 | 1.727 | -0.0792 | No |
| 5 | SCRIB |  |  | 4562 | 1.299 | -0.1416 | No |
| 6 | BCL6 |  |  | 5145 | 1.099 | -0.1678 | No |
| 7 | NHLH2 |  |  | 5603 | 0.943 | -0.1870 | No |
| 8 | MDM2 |  |  | 5816 | 0.872 | -0.1890 | No |
| 9 | MAN2B1 |  |  | 5858 | 0.857 | -0.1783 | No |
| 10 | FEZ1 |  |  | 6211 | 0.747 | -0.1928 | No |
| 11 | SMTN |  |  | 6675 | 0.598 | -0.2181 | No |
| 12 | LCAT |  |  | 7341 | 0.369 | -0.2622 | No |
| 13 | ENG |  |  | 7369 | 0.359 | -0.2585 | No |
| 14 | SURF1 |  |  | 7829 | 0.178 | -0.2902 | No |
| 15 | FHL2 |  |  | 8959 | -0.292 | -0.3705 | No |
| 16 | BAX |  |  | 8974 | -0.302 | -0.3667 | No |
| 17 | GADD45A |  |  | 8982 | -0.305 | -0.3623 | No |
| 18 | PMAIP1 |  |  | 9321 | -0.468 | -0.3803 | No |
| 19 | BBC3 |  |  | 9752 | -0.695 | -0.4015 | No |
| 20 | DGKA |  |  | 9842 | -0.751 | -0.3961 | No |
| 21 | APLP1 |  |  | 9894 | -0.779 | -0.3875 | No |
| 22 | ZNF7 |  |  | 9905 | -0.787 | -0.3756 | No |
| 23 | PLK3 |  |  | 10654 | -1.284 | -0.4113 | Yes |
| 24 | DDB2 |  |  | 10765 | -1.362 | -0.3977 | Yes |
| 25 | CDKN1A |  |  | 10787 | -1.380 | -0.3772 | Yes |
| 26 | CSPG2 |  |  | 11207 | -1.748 | -0.3807 | Yes |
| 27 | CES2 |  |  | 11303 | -1.826 | -0.3585 | Yes |
| 28 | CTDSP2 |  |  | 11625 | -2.146 | -0.3482 | Yes |
| 29 | IGFBP6 |  |  | 12398 | -3.098 | -0.3566 | Yes |
| 30 | NDN |  |  | 12435 | -3.157 | -0.3087 | Yes |
| 31 | KIAA0247 |  |  | 12655 | -3.533 | -0.2685 | Yes |
| 32 | ABCA3 |  |  | 13025 | -4.531 | -0.2236 | Yes |
| 33 | BTG2 |  |  | 13039 | -4.613 | -0.1506 | Yes |
| 34 | NINJ1 |  |  | 13069 | -4.720 | -0.0770 | Yes |
| 35 | SCGB2A2 |  |  | 13251 | -5.975 | 0.0052 | Yes |
Table: GSEA details [plain text format]

  

Fig 2: KANNAN\_P53\_UP: Random ES distribution      
 Gene set null distribution of ES for **KANNAN\_P53\_UP**
